# Validation of a routine two‐sample iohexol plasma clearance assessment of GFR and an evaluation of common endogenous markers in a rat model of CKD

**DOI:** 10.14814/phy2.13205

**Published:** 2017-05-08

**Authors:** Mandy E. Turner, Kimberly J. Laverty, Paul S. Jeronimo, Martin Kaufmann, Glenville Jones, Christine A. White, Rachel M. Holden, Michael A. Adams

**Affiliations:** ^1^ Department of Biomedical and Molecular SciencesFaculty of Health SciencesQueen's UniversityKingstonOntarioCanada; ^2^ Department of MedicineFaculty of Health SciencesQueen's UniversityKingstonOntarioCanada

**Keywords:** Creatinine, GFR, inulin, iohexol, plasma clearance

## Abstract

Endogenous markers of kidney function are insensitive to early declines in glomerular filtration rate (GFR) and in rodent models, validated, practical alternatives are unavailable. In this study, we determined GFR by modeling the plasma clearance of two compounds, iohexol and inulin, and compared the findings to common endogenous markers. All plasma clearance methods of both iohexol and inulin detected a decline in renal function weeks prior to any increase in endogenous marker. Iohexol plasma clearance and inulin plasma clearance had a very high agreement and minimal bias when using 12‐sample models. However, only iohexol could be accurately simplified to a two‐sample, one‐compartment estimation strategy. Following an IV injection of low‐dose iohexol and two timed blood samples at 30 and 90 min, one can accurately approximate a complex 12‐sample strategy of plasma clearance. This method is simple enough to use in routine, longitudinal analysis of larger cohort animal studies.

## Introduction

The delayed rise in plasma creatinine or urea is well known to result in an underestimation of declines in kidney function, particularly at early stages (Morgan et al. 1978; Levey 1990; Katayama et al. 2011). Despite their limitations, endogenous biomarkers of kidney function, such as creatinine and urea, are widely used to evaluate kidney function in experimental models. The gold standard methodology for measuring glomerular filtration rate (GFR), both in humans and in animal models, is the urinary clearance of inulin (Levey 1990; Shobeiri et al. 2013). This method is rarely used clinically (White et al. 2008; Eknoyan et al. 2012) and experimentally, likely due to the technical difficulty, cost, or feasibility of use in repeated measures.

Plasma clearance (PC) of an exogenous substance to estimate GFR is much simpler than urinary clearance and is more accurate than endogenous biomarkers (Brøchner‐Mortensen 1985). Following a single injection of exogenous markers (such as iohexol or inulin), plasma clearance can be determined from the decline in plasma concentration over time (i.e. the, area under the curve [AUC]) and the dose (D) injected (PC = D/AUC) (Peters 2003). However, the accuracy of PC is highly dependent on both the timing and the number of sample points used to define the area under the curve (AUC) (Peters 2003; Murray et al. 2013). To provide an alternative to creatinine and urea in experimental research, plasma clearance method needs to be more practical as well as accurate. Iohexol is an inexpensive and readily available marker that has been used clinically to evaluate GFR (Soveri et al. 2014), however, the ideal protocol has yet to be established for rat models.

Using an longitudinal adenine‐induced model of CKD, this study sought to (i) compare GFR measurements using inulin and iohexol PC to commonly used endogenous markers (creatinine, urea, and cystatin‐C); (ii) determine the impact of sample timing and model choice on the accuracy of the PC of iohexol and inulin; and (iii) investigate how a reduction in the number of samples used in estimation would affect accuracy.

## Materials and Methods

### Literature search

We wanted to examine the prevalence of various renal function assessments in recent experimental rat studies. A computer bibliographic search of the OVID MEDLINE database was done to identify potentially relevant articles. The search included four search terms: ‘acute kidney injury’, or ‘chronic renal insufficiency’, or ‘renal hypertension’, and ‘rats’, limited to English‐language papers published from 2014 to present (July 4, 2016). This search returned 571 hits. The following were excluded: 55 were either repeats or nonaccessible, 21 were review articles, and 102 did not measure kidney function. The literature review is based on the remaining 392 research articles.

### Experimental animals and GFR assessment

Male Sprague–Dawley rats (*N* = 8: Charles River, Quebec) were aged 11–13 weeks and weighed 400–475 g at the experimental start. Progressive kidney disease was induced using a previously described 0.25% adenine diet over 5 weeks (Shobeiri et al. 2013). All experiments were performed in accordance with the Canadian Council on Animal Care and approved by Queen's University Animal Care Committee. Before commencement of the adenine diet and each subsequent week (5 weeks), the kidney function of each rat was evaluated via a standard protocol. Under light anesthesia with isoflurane (5% induction, 1.5% maintenance in 1 L/min O_2_), dialyzed fluorescein isothiocyanate (FITC)‐tagged inulin solution (Sigma Aldrich, 2.5 *μ*L/kg of BW) and iohexol (Omnipaque 300; 25.9 mg/kg of BW), separated by 200 *μ*L of 50 *μ*IU/mL of heparinized saline, were serially injected into the tail vein to ensure 100% bioavailability. Preliminary studies showed no impact of serial injection or order of injection on clearance. Following injection, rats were immediately removed from anesthesia and gained full consciousness within 5 min. Saphenous vein blood samples of 140 *μ*L via heparinized capillary tubes were taken at 12 time points following injection: 0, 2, 5, 10, 20, 30, 60, 90, 120,180, 240, and 300 min.

### Preparation and quantification of FITC‐Inulin

Preparation and measurement of FITC‐Inulin solution were modified from Qi et al. (2004). A 5% FITC‐inulin solution was made by dissolving 0.5 g of FITC‐Inulin (Sigma‐Aldrich) in 10 mL of 0.85% sodium chloride solution. In order to achieve full solubility, the solution is heated to at most 80°C, higher temperatures compromise stability. To remove residual unbound FITC, the solution was dialyzed in 1.8 L of 0.85% sodium chloride at room temperature for 24 h using 1 kDa dialysis membrane (Puralyzer Mega 1000 Dialysis Kit with 10–15 mL cap) as per instructions. Due to the risk of photobleaching with FITC‐inulin, the dialysis apparatus, as well as all storage containers for the FITC‐inulin solution were shielded from light. The new concentration (mg/mL) of FITC‐inulin was calculated based on the volume change in the dialysate and stored at 4°C. Each week, a new solution of FITC‐inulin was made and the concentration of the solution was 39.2 ± 2.80 mg/mL. The solution was sterilized by passage through a 0.20 *μ*m filter (Filtropur) directly before experimental use. Dose of inulin was determined by measuring sterile filtrate on the assay described below, such that if the sterile filtration effected dose concentration, it would be accounted for. FITC‐inulin concentrations were quantified in rat plasma using fluorescence relative to serial dilutions of a known concentration. The standard curve consisted of 10 dilutions of the stock dialyzed solution from 1:200 to 1:100,000. As pH significantly affects fluorescence (Lorenz and Gruenstein 1999), plasma samples were buffered to 7.4 using 500 mmol/L HEPES (Sigma‐Aldrich). Titrated samples and standards were loaded on a 96‐well flat bottom solid black plate and measured using a 96‐well plate fluorometer (BIO‐TEK KC4V3.3, Vermont) with 485‐nm excitation and read at 528‐nm emission. In each standard well, there was 60 *μ*L of 500 mmol/L HEPES, 12 *μ*L of standard, and 12 *μ*L of control rat plasma. In each sample well: 60 *μ*L of 500 mmol/L HEPES, 12 *μ*L of sample rat plasma, and 12 *μ*L of 0.85% sodium chloride solution. All samples and standards were run in duplicate. A linear relationship with a regression coefficient of 0.999 between fluorescence counts and inulin concentration was achieved.

### Quantification of iohexol

A 10‐point calibrator (1–400 *μ*g/mL) was generated in‐house by spiking synthetic iohexol (Toronto Research Chemicals) into pooled human serum. Samples were extracted using a modification of the method of Annesley and Clayton (2009), where 20 *μ*L of calibrator or rat plasma was diluted with 430 *μ*L of water in microcentrifuge tubes. Protein precipitation was carried out by adding 100 *μ*L of 0.2 mol/L zinc sulfate, 450 *μ*L of methanol, and 1 *μ*g of penta‐deuterated (d5) iohexol internal standard (Toronto Research Chemicals), and mixed by vortexing after addition of each component. Extracts were centrifuged at 12,000***g*** for 10 min, and 125 *μ*L of supernatant was diluted into LC‐MS vials containing 875 *μ*L of 10% (v/v) methanol/water.

LC‐MS/MS analysis was conducted using Waters ACQUITY UPLC/Xevo TQ‐S instruments, where mobile phase A and B comprised methanol and water (Optima, Fisher), each supplemented with 0.1% (v/v) glacial acetic acid (Sigma Aldrich). Duplicate 10 *μ*L injections were analyzed for each sample. Separation was conducted using a linear gradient elution of 5–80% mobile phase B over 3 min at 400 *μ*L/min, on a Waters HSS‐T3 column (1.7 *μ*m; 2.1 × 100 mm) maintained at 40°C. Iohexol and iohexol‐d5 internal standard were detected in electrospray positive mode using MRM transitions *m/z* 822.0→804.1 and *m/z* 827.0→809.1 with the following optimized settings: capillary; 3 kV, cone: 64 V, collision; 17 eV.

Calibrators were aligned to the LC‐MS/MS method of the University of Minnesota Medical Center‐Fairview via sample exchange. Seven quality control samples ranging from 5 to 400 *μ*g/mL iohexol were analyzed up to three times on each of 12 days. Intraassay CVs ranged from 5 to 7%, and interassay CVs ranged from 2 to 8%. Iohexol concentration in quality control samples exhibited a mean bias of −0.8% (range −6 to 5%) as compared to University of Minnesota reference values, with a lower limit of quantification of 0.2–0.5 *μ*g/mL. Iohexol can also be quantified using high‐pressure liquid chromatography (Krutzén et al. 1985), iodine quantification (Bäck et al. 1988), and X‐ray fluorescence (Grönberg et al. 1981).

### Endogenous marker quantification

Creatinine was evaluated via the Jaffe method and urea via a spectrophotometric assay (QuantiChrom^™^ Creatinine/Urea Assay Kit, Bioassay Systems). Cystatin‐C was quantified using an ELISA (Quantikine^®^ ELISA, Mouse/Rat Cystatin C Immunoassay, R&D systems).

### Pharmacokinetic analysis

PC was calculated by dividing dose (ng) by the estimated area under the curve.

#### Trapezoidal approximation

**Figure 1:**
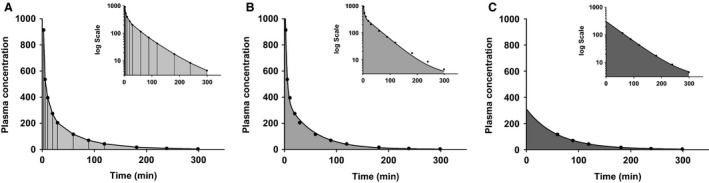
Plasma clearance models. (A) Trapezoidal approximation of the area under the curve. This method is not dependent on modeling but the accuracy is highly dependent on the number of sample points. (B) Two‐compartment model which considers both the alpha (equilibration of the marker with extracellular fluid) and the beta (renal clearance) phases. (C) One‐compartment model is the simplest of the models, but considers the alpha phase to be negligible. The figure indicates data of inulin plasma clearance of animal A8 at 3 weeks of adenine diet.

For this method, the sum of all trapezoids generated across the sample time points (minus background) was used (Chiou 1978). If the concentration of the marker had not declined sufficiently (<0.5 *μ*g/mL), the remaining section was determined by a log‐linear extrapolation to 0.5 *μ*g/mL, as required (Fig. 1A)

#### Two‐compartment model

This model considers the alpha and beta phase of clearance and is modeled by a biexponential decay with the plateau set to 0 *μ*g/mL (Fig. 1B) (Peters 2003)

$$\text{Plasma concentration =}\;Ae^{-\alpha t}+Be^{-\beta t}$$

Thus, the PC is calculated as

$$\text{PC}=\frac{\text{DOSE}}{\frac{A}{\alpha }+\frac{B}{\beta }}$$

#### One‐compartment model

This model only considers points after the first phase of clearance has ended (Fig. 4C) (Peters 2003). The log‐linear slope during the beta phase begins to change at 30 min (Table S1), so only time points from 30 min onward were included. This is modeled as a single‐exponential decay with the plateau set to zero (Fig. 1C).

$$\text{Plasma concentration =}Be^{-\beta t}$$

Thus, PC is calculated as

$$\text{PC}=\frac{\text{DOSE}}{\frac{B}{\beta }}$$

#### Two‐sample one‐compartment model

The following derived formula was used to calculate the PC from two samples where *I* = dose (*μ*g), *c* = concentration (*μ*g/mL), and *t* = time from injection the sample was taken:

$$\text{Plasma Clearance}=I\times \frac{\text{ln}\left(\frac{C_{2}}{C1}\right)}{t_{1}-t_{2}}\times {\left(\frac{C_{1}^{t2}}{C_{2}^{t1}}\right)}^{\frac{1}{t_{1}-t_{2}}}$$

Statistics were performed using GraphPad Prism (V6).

### Analysis

Weekly PC values and endogenous plasma concentrations of creatinine, urea, and cystatin‐C were compared to week 0 using a repeated measures one‐way ANOVA to evaluate the ability of each biomarker to detect an early change in GFR.

**Figure 2:**
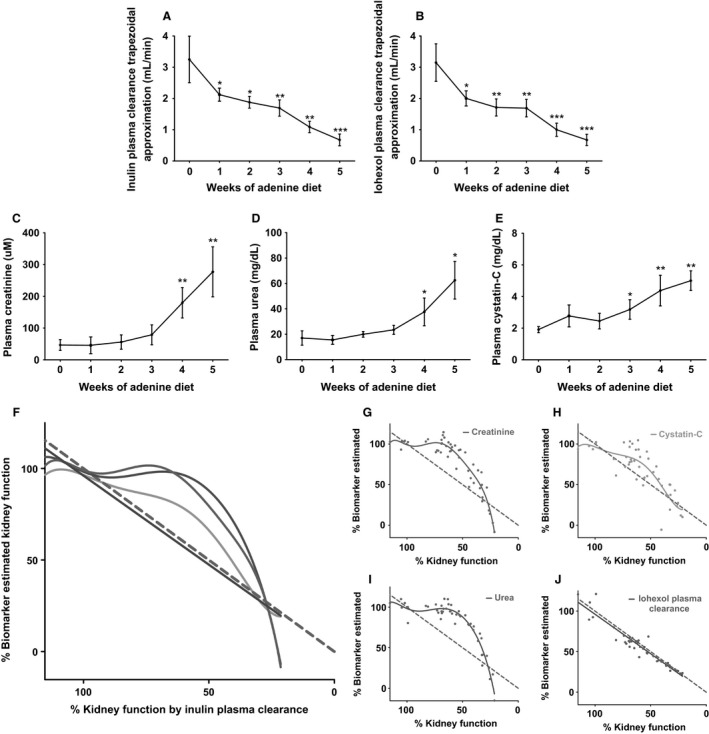
Sensitivity and normalization of kidney function measurements. (A–E) Change in trapezoidal plasma clearance of both iohexol and inulin and endogenous biomarkers: plasma creatinine, urea, and cystatin‐C by week of adenine diet‐induced kidney dysfunction. Results analyzed using a repeated measures one‐way ANOVA with post hoc paired *T*‐tests with a Tukey correction on the *P*‐value comparing each week to week 0 (*P <* *0.05, **P < 0.001, ***P < 0.0001). Error bars show SD. (F–J) The kidney function estimated by inulin plasma clearance as a reference method and the % estimated by the biomarker or iohexol were plotted against each other. An *F*‐test was employed to determine whether a straight line or sixth‐order polynomial fit the data better and the result was plotted. Iohexol plasma clearance fit a straight line. Creatinine, urea, and cystatin‐C fit to a sixth‐order polynomial. Gray dotted line indicates inulin plasma clearance plotted against itself – unity.

In order to visualize the predictive value of each endogenous biomarker and iohexol plasma clearance, each marker of kidney function was normalized to inulin trapezoidal PC (Fig. 2F–J). Each point on the graph is representative of one animal at each week during the study. The mean value at week 0 of each biomarker was defined to be representative of 100% kidney function. Using trapezoidal approximation of inulin plasma clearance as the reference method, all individual inulin data points were evaluated as a percent of that value (115.4–23.8%). Each endogenous biomarker and iohexol plasma clearance (trapezoidal) were assumed to be representative of 100–23.8% of kidney function, an estimation based on inulin plasma clearance. The kidney function estimated by inulin plasma clearance as a reference method and the % estimated by the biomarker or iohexol were plotted against each other. An *F*‐test was employed to determine whether a straight line or sixth‐order polynomial fit the data better and the result was plotted.

Inulin plasma clearance was chosen as a reference measure as in a rat (Sturgeon et al. 1998) and mouse models (Qi et al. 2004), and inulin PC via a two‐compartment model exhibited high agreement with inulin urinary clearance with minimal bias. The trapezoidal approximation of PC is used as a reference method because this approach is not prone to modeling errors, and the large number and high density of samples used in this study make it very accurate.

Linear regressions were done between all PC models of both inulin and iohexol to evaluate agreement. Bias or absolute bias was calculated as reference method‐comparative method. Precision is reported as the standard deviation of bias. The % bias is calculated by dividing the absolute bias by the mean of the two measures. Accuracy is the percentage of sample values with a % bias of <10 or 15.

## Results

An OvidMedline search of the last 2 years (2014–current) revealed 392 published studies involving rats filed under the MeSH headings “acute kidney injury”, “chronic kidney disease”, and “hypertension‐induced nephropathy”. Of these 392 studies, the majority (92%) assessed and reported kidney function using endogenous markers, such as creatinine (blood or urinary) or urea, exclusively. Most (52%) based their assessment only on serum or plasma measurements. Thirteen percent measured only blood creatinine or urea, 34% measured both, and 5% included analyses of cystatin‐C, uric acid, and the nitrate‐to‐nitrite ratio. Thirty‐nine percent of studies supplemented serum and plasma endogenous markers with urine analysis of urea, protein, or creatinine, including the calculation of creatinine clearance. Of the 392 studies, 27 (6.8%) of the published studies in this literature search used exogenous markers to determine GFR. Twenty‐one of these studies used constant infusion of inulin followed by measurements of urinary clearance. Other methods included urinary clearance of infused ^51^Cr‐EDTA and plasma clearance of inulin or ^99^Tc‐DPTA after a single injection. No studies have used iohexol clearance as a measure of renal function to evaluate study outcomes.

### Endogenous marker and PC profiles as kidney function declines

The profile of the weekly change in each biomarker as CKD was generated over 5 weeks is presented in Figure 2. Using trapezoidal modeling, the PC of both inulin (Fig. 2A) and iohexol (Fig. 2B) was significantly decreased after 1 week of the adenine diet. In contrast, plasma creatinine (Fig. 2C) and plasma urea (Fig. 2D) levels did not become significantly elevated until week 4 of the adenine diet. A significant increase in plasma cystatin‐C elevated was observed by week 3 (Fig. 2E).

Iohexol PC and levels of creatinine, urea, and cystatin‐C were compared to gold standard trapezoidal inulin clearance (Fig. 2F–J). Iohexol plasma clearance was very similar to inulin plasma clearance (Fig. 2J). In contrast, changes in creatinine, urea, and cystatin‐C were not evident until kidney function had dropped by nearly 40–50% (Fig. 2G–I). Evident from Figure 2H, cystatin‐C exhibited higher agreement with inulin trapezoidal plasma clearance on average, however, displayed more variability.

### Comparison of iohexol and inulin PC using trapezoidal, two‐compartment, and one‐compartment models

Comparative values between all PC metrics and their biases are presented in Table 1. On average, the iohexol trapezoidal model had low bias (0.04 mL/min), high precision (0.23), and a high 15% accuracy (85%) as compared to gold standard inulin trapezoidal. The linear regression coefficient between the two was *R* ^2^ = 0.97.

**Table 1:** Linear regression coefficients, mean bias, precision, and accuracy comparing all plasma clearance measures of both inulin and iohexol

Reference measure	Comparative measure	*R* ^2^	Bias/Precision^a^ (mL/min)	%Bias/Precision^b^	10% Accuracy^c^	15% Accuracy^c^
Inulin trapezoidal	Iohexol two‐compartment	0.93	−0.02 ± 0.37	−1.2 ± 16.4	55.8	69.8
	Iohexol trapezoidal	0.97	0.04 ± 0.23	3.8 ± 11.6	73.9	82.6
	Iohexol one‐compartment	0.96	0.02 ± 0.28	0.12 ± 13.24	56.5	80.4
	Inulin two‐compartment	0.97	−0.28 ± 0.25	−19.9 ± 7.51	4.3	21.9
	Inulin one‐compartment	0.98	−0.57 ± 0.37	−26.6 ± 7.3	0	2.17
Inulin two‐compartment	Iohexol two‐compartment	0.93	0.35 ± 0.35	18.5 ± 12.6	16.3	37.2
	Iohexol trapezoidal	0.97	0.41 ± 0.25	23.3 ± 9.9	6.5	17.4
	Iohexol one‐compartment	0.96	0.39 ± 0.31	19.7 ± 10.1	13.0	37.0
	Inulin one‐compartment	0.99	−0.49 ± 0.18	−6.81 ± 5.14	74.5	91.5
Iohexol trapezoidal	Iohexol two‐compartment	0.97	−0.07 ± 0.19	−5.5 ± 9.5	74.4	83.7
	Iohexol one‐compartment	0.98	−0.02 ± 0.13	−3.7 ± 8.0	78.3	87.0
Iohexol two‐compartment	Iohexol one‐compartment	0.98	0.03 ± 0.16	0.9 ± 7.0	95.3	95.3

Each animal (*N* = 8) at each evaluation (*N* = 6) is an individual data point (*N* = 44–48). Linear regression coefficients (*R* ^2^) weighted 1/*C* ^2^.

a Bias or absolute bias is calculated as reference method‐comparative method. Precision is the standard deviation of bias.

b % Bias is calculated by dividing the absolute bias by the mean of the two measures.

c Accuracy is the percentage of sample values with a % bias of <10 or 15.

**Figure 3:**
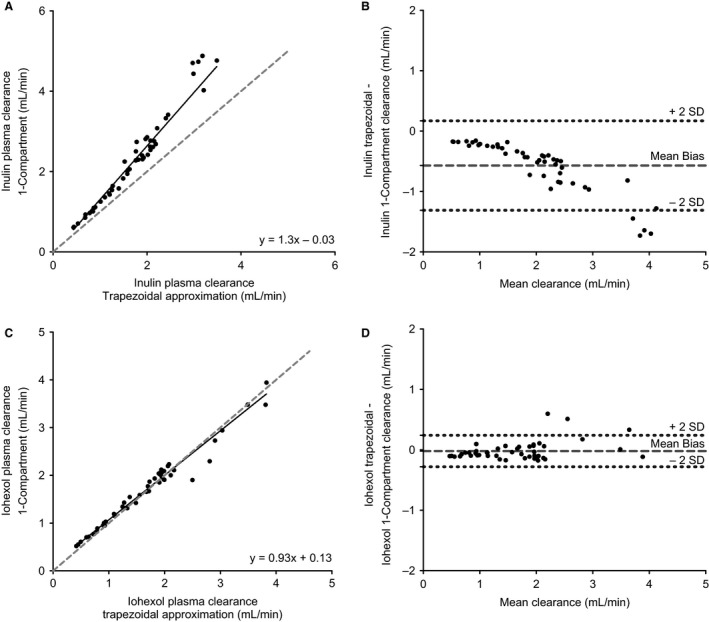
Comparison of inulin and iohexol trapezoidal plasma clearance to simplified one‐compartment model. (A) Plot of inulin plasma clearance as measured by trapezoidal approximation plotted against the one‐compartment model estimation. The gray dotted line indicates unity. (B) Bland–Altman plot of absolute bias of inulin one‐compartment model compared to the reference measure of inulin trapezoidal plasma clearance plotted against the mean of both clearances. The center line indicates the mean bias of −0.57 mL/min with the dotted line indicating the 95% confidence interval or 2 SD (±0.74 mL/min). (C) Plot of iohexol plasma clearance as measured by trapezoidal approximation plotted against the one‐compartment model estimation. The gray dotted line indicates unity. (D) Bland–Altman plot of absolute bias of iohexol one‐compartment model compared to the reference measure of iohexol trapezoidal plasma clearance plotted against the mean of both clearances. The center line indicates the mean bias of −0.02 mL/min with the dotted line indicating the 95% confidence interval or 2 SD (±0.26).

The trapezoidal approximation was compared to the one‐ and two‐compartment models for both inulin and iohexol plasma clearance. For inulin, despite the strong correlation with its trapezoidal approximation counterpart, the one‐compartment (*R* ^2^ = 0.98) and two‐compartment (*R* ^2^ = 0.97) models introduced large negative biases (−26.6 ± 7.3%, −19.9 ± 7.51%) which became worse at a higher GFR (Fig. 3B). Furthermore, <5% of the measurements fell within 10% of the reference measure with either of the models (Table 1).

In contrast, with iohexol, there was less impact on the one‐ and two‐compartment models on the determination of kidney function (Table 1, Fig. 2C,D). Specifically, both the one‐ and two‐compartment models had high degree of agreement (*R* ^2^ = 0.98, 0.97) and much less bias (−3.7 ± 8.0%, −5.5 ± 9.5%) compared to the trapezoidal method, and the majority of measurements fell within 10% of the reference measure for both models (78.3%, 74.4%).

### Simplifying the one‐compartment method to two‐sample time points

**Figure 4:**
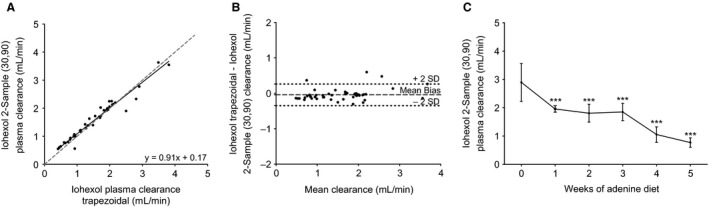
Iohexol plasma clearance calculated by two blood samples at 30 and 90 min. (A) Plot of iohexol two‐sample plasma clearance (30, 90) against the full 12‐sample trapezoidal approximation. The gray dotted line indicates unity. (B) Bland–Altman plot of absolute bias of two‐sample iohexol plasma clearance compared to the reference measure of iohexol trapezoidal plasma clearance plotted against the mean of both clearances. The center line indicates the mean bias of −0.04 mL/min with the dotted line indicating the 95% confidence interval or 2 SD (±0.30 mL/min). (C) Sensitivity of two‐sample iohexol plasma clearance (30, 90) to change by the week of adenine diet‐induced kidney dysfunction. Results analyzed using a one‐way ANOVA with post hoc *T*‐tests with a Tukey correction on the *P*‐value comparing each week to week 0 (***P < 0.0001). Error bars show SD.

Given that the alpha phase of PC was determined to be complete by 30 min only, values between 30 and 120 min were used for the two‐point one‐compartment assessment of kidney function (Table S1). Analysis using the 30‐ and 90‐min time points had the overall, strongest agreement (*R* ^2^ = 0.94–0.97), and accuracy regardless of the method of assessment (Table 2). Figure 4(A–B) shows this two‐sample assessment yielded very similar GFRs to the trapezoidal full dataset, having negligible bias (−0.04 ± 0.15 mL/min). There was also a similar decline in GFR, decreasing significantly 1 week after starting the adenine diet (Fig. 4C). In contrast, no simplification of this type was possible using inulin PC, as the one‐compartment model introduced such a large bias.

**Table 2:** Two‐sample permutations of iohexol one‐compartment plasma clearance compared to iohexol plasma clearance calculated by trapezoidal, one‐, and two‐compartment models – linear regression coefficients, bias, precision, and accuracy

Sample times	Iohexol one‐compartment			Iohexol trapezoidal			Iohexol two‐compartment		
	*R* ^2^	Bias/Precision^a^	10% Ac^b^	*R* ^2^	Bias/Precision^a^	10% Ac^b^	*R* ^2^	Bias/Precision^a^	10% Ac^b^
30, 60	0.87	0.02 ± 0.27	72.7	0.86	0.00 ± 0.32	50.0	0.84	0.04 ± 2.79	69.0
60, 90	0.90	0.18 ± 0.95	76.7	0.92	0.16 ± 0.94	47.7	0.91	0.19 ± 0.95	72.5
90, 120	0.43	0.15 ± 0.37	73.3	0.73	0.13 ± 0.43	51.1	0.66	0.12 ± 0.42	66.7
30, 90	0.97	−0.02 ± 0.12	91.1	0.96	−0.04 ± 0.15	66.7	0.94	0.01 ± 0.18	83.3
60, 120	0.82	−0.04 ± 0.23	53.3	0.91	−0.06 ± 0.19	66.7	0.95	−0.01 ± 0.32	54.8

Each animal (*N* = 8) at each evaluation (*N* = 6) is an individual data point (*N* = 44–48). Linear regression coefficients (*R* ^2^) weighted 1/*C* ^2^.

a Bias or absolute bias is calculated as reference method – two‐sample clearance. Precision is the standard deviation of bias.

b 10% Ac or 10% Accuracy is the percentage of sample values with a % bias of <10. % Bias is calculated by dividing the absolute bias by the mean of the two measures.

## Discussion

Plasma clearance (PC) of either iohexol or inulin, regardless of the model, detected a significant decline in kidney function weeks before any of the endogenous markers. Plasma creatinine or plasma urea did not significantly change despite inulin‐based assessments indicating that GFR had dropped by ~50%. Although plasma cystatin‐C was found to have a higher agreement with the gold standard than the other endogenous markers, the high degree of variability would still limit its utility, particularly as it has been shown to increase during periods of acute inflammation (Deyà‐Martínez et al. 2016). Despite this serious limitation of endogenous biomarkers for assessment of kidney function, our literature search revealed that 93% of papers exclusively rely on these markers. These results, together, suggest that many studies may be at risk for underestimating or missing significant kidney dysfunction.

The relationship of the endogenous markers with plasma clearance reflects a trend that is appreciated in clinical practice, due to the kinetics of a constantly produced marker: the delayed rise of endogenous markers early on in kidney dysfunction and then exacerbated rise as kidney function worsens. In rat models of kidney decline, evaluations of kidney dysfunction are far less standardized and limitations of endogenous markers may be more easily overlooked.

Inulin and iohexol showed high agreement with each other using the models that encompassed all 12 time points (trapezoidal and two‐compartment). This suggests that iohexol plasma clearance using 12‐sample points characterizes kidney function as accurately as validated reference measures of inulin plasma clearance. A limitation of this study is that we did not compare iohexol plasma clearance to urinary clearance of inulin. Plasma clearance of inulin was chosen as it has been shown to have high agreement with urinary clearance (Sturgeon et al. 1998; Qi et al. 2004) and allows to compare longitudinal evaluations, as urinary clearance is often terminal.

A one‐compartment model assumes that the first phase of distribution of a substance into the extracellular fluid is negligible. The results of our study show that this is an inappropriate assumption for inulin PC as the simplification to a one‐compartment model introduced a large bias. Furthermore, in conditions with significant variation in extracellular fluid content (e.g., obese strains), the impact on accuracy may be even greater, although this has not been shown. Iohexol displayed a different pharmacokinetic profile such that simplification to a one‐compartment model introduced minimal bias. Inulin is a large (2000–5000 g/mol) polysaccharide chain, whereas iohexol is relatively small (821 g/mol) iodinated molecule. Although full pharmacokinetic characterization and comparison of the two molecules have not been completed, it is likely that the differences will be related to factors such as the size of the molecules and the rate of equilibration with extracellular fluid.

A previous study was completed in rats validating iohexol plasma clearance which yielded fair, although poorer, results with the agreement of iohexol and inulin measures of GFR (Passos et al. 2015). This study used only four samples in total using a two‐compartment model of clearance. The methodology involved an intraperitoneal injection of iohexol, which has lower bioavailability, and a longer period of equilibration, making the plasma clearance less suitable for one‐compartment modeling. A tail vein injection, used in this study can be done without anesthesia and has much higher availability and as the results indicated, negligible equilibration time making this method more suitable for one‐compartment modeling. A two‐sample protocol, such as the one suggested here, using iohexol, has been proposed in humans (Ng et al. 2011).

An important finding of this study was that the two‐sample iohexol method (30 and 90 min) closely approximated the 12‐sample clearance models of iohexol with very high accuracy, and almost no bias and minimal loss of precision. It was determined that the first sample time must be obtained after the first compartment of clearance (in this model 30 min+) and the second sample needs to be taken when there are still measurable marker concentrations. For example, the 30 and 120 time points yielded less accurate GFR estimation, which may be due to low marker concentrations, where measurement error will have an even greater impact on GFR estimations due to the log‐linear relationship.

In conclusion, this study demonstrated that a simplified two‐sample determination of iohexol PC is feasible and accurate and could be employed in large‐scale animal studies that require measurement of kidney function. The findings also reinforce the marked lack of accuracy of endogenous markers to detect mild‐to‐moderate declines in kidney function.

## Conflict of Interest

Through a Queen's University/Waters Corporation research contract, Waters has generously provided the LC‐MS/MS instrumentation used in this study.

## Data Accessibility

## Supporting information




**Table S1.** Log‐linear slope for determining sample time points for one‐compartment analysis.

## References

[phy213205-bib-0001] Annesley, T. M. , and L. T. Clayton . 2009 Ultraperformance liquid chromatography‐tandem mass spectrometry assay for iohexol in human serum. Clin. Chem. 55:1196–1202. 19359533 10.1373/clinchem.2008.121533

[phy213205-bib-0002] Bäck, S. E. , P. Masson , and P. Nilsson‐Ehle . 1988 A simple chemical method for the quantification of the contrast agent iohexol, applicable to glomerular filtration rate measurements. Scand. J. Clin. Lab. Invest. 48:825–829. 3238327 10.3109/00365518809088767

[phy213205-bib-0003] Brøchner‐Mortensen, J. 1985 Current status on assessment and measurement of glomerular filtration rate. Clin. Physiol. 5:1–17. 10.1111/j.1475-097x.1985.tb00742.x 3882316

[phy213205-bib-0004] Chiou, W. L. 1978 Critical evaluation of the potential error in pharmacokinetic studies of using the linear trapezoidal rule method for the calculation of the area under the plasma level–time curve. J. Pharmacokinet. Biopharm. 6:539–546. 731416 10.1007/BF01062108

[phy213205-bib-0005] Deyà‐Martínez, À. , C. Fortuny , P. Soler‐Palacín , O. Neth , E. Sánchez , A. Martín‐Nalda , et al. 2016 Cystatin C: a marker for inflammation and renal function among HIV‐infected children and adolescents. Pediatr. Infect. Dis. J. 35:196–200. 26479972 10.1097/INF.0000000000000960

[phy213205-bib-0006] Eknoyan, G. , N. Lameire , K. U. Eckardt , B. L. Kasiske , D. C. Wheeler , A. Levin , et al. 2012 clinical practice guideline for the evaluation and managment of chronic kidney disease. Kidney Int. 5–14:2013.

[phy213205-bib-0007] Grönberg, T. , T. Almén , K. Golman , K. Lidén , S. Mattsson , and S. Sjöberg . 1981 Non‐invasive estimation of kidney function by x‐ray fluorescence analysis. Method for in vivo measurements of iodine‐containing contrast media in rabbits. Phys. Med. Biol. 26:501–506. 7243881 10.1088/0031-9155/26/3/012

[phy213205-bib-0008] Katayama, R. , K. Watanabe , N. Yamagishi , S. Abe , H. Satoh , and K. Furuhama . 2011 Sequential measurements of glomerular filtration rate in conscious rats by a bolus injection of iodixanol and a single blood sample: sequential measurement of GFR in rats. J. Appl. Toxicol. 31:360–365. 21259291 10.1002/jat.1619

[phy213205-bib-0009] Krutzén, E. , S. E. Bäck , I. Nilsson‐Ehle , and P. Nilsson‐Ehle . 1985 Plasma clearance of a new contrast agent, iohexol: a method for the assessment of glomerular filtration rate. J. Lab. Clin. Sci. 104:955–961. 6438261

[phy213205-bib-0010] Levey, A. S. 1990 Measurement of renal function in chronic renal disease. Kidney Int. 38:167–184. 2200925 10.1038/ki.1990.182

[phy213205-bib-0011] Lorenz, J. N. , and E. Gruenstein . 1999 A simple, nonradioactive method for evaluating single‐nephron filtration rate using FITC‐inulin. Am. J. Physiol. 276:F172–F177. 9887093 10.1152/ajprenal.1999.276.1.F172

[phy213205-bib-0012] Morgan, D. B. , S. Dillon , and R. B. Payne . 1978 The assessment of glomerular function: creatinine clearance or plasma creatinine? Postgrad. Med. J. 54:302–310. 673986 10.1136/pgmj.54.631.302 PMC2425131

[phy213205-bib-0013] Murray, A. W. , M. C. Barnfield , M. L. Waller , T. Telford , and A. M. Peters . 2013 Assessment of glomerular filtration rate measurement with plasma sampling: a technical review. J. Nucl. Med. Technol. 41:67–75. 23658207 10.2967/jnmt.113.121004

[phy213205-bib-0014] Ng, D. K. , G. J. Schwartz , L. P. Jacobson , F. J. Palella , J. B. Margolick , B. A. Warady , et al. 2011 Universal GFR determination based on two time points during plasma iohexol disappearance. Kidney Int. 80:423–430. 21654718 10.1038/ki.2011.155 PMC3146568

[phy213205-bib-0015] Passos, M. T. , S. K. Nishida , N. O. Câmara , M. H. Shimizu , and G. Mastroianni‐Kirsztajn . 2015 Iohexol clearance for determination of glomerular filtration rate in rats induced to acute renal failure [Online]. PLoS ONE 10:1–8. 10.1371/journal.pone.0123753 PMC4395274 25875005

[phy213205-bib-0016] Peters, A. M. 2003 The kinetic basis of glomerular filtration rate measurement and new concepts of indexation to body size. Eur. J. Nucl. Med. Mol. Imaging 31:137–149. 14593500 10.1007/s00259-003-1341-8

[phy213205-bib-0017] Qi, Z. , I. Whitt , A. Mehta , J. Jin , M. Zhao , R. C. Harris et al. 2004 Serial determination of glomerular filtration rate in conscious mice using FITC‐inulin clearance. AJP Ren. Physiol. 286:F590–F596. 10.1152/ajprenal.00324.2003 14600035

[phy213205-bib-0018] Shobeiri, N. , J. Pang , M. A. Adams , and R. M. Holden . 2013 Cardiovascular disease in an adenine‐induced model of chronic kidney disease: the temporal link between vascular calcification and haemodynamic consequences. J. Hypertens. 31:160–168. 23183279 10.1097/HJH.0b013e32835b15bb

[phy213205-bib-0019] Soveri, I. , U. B. Berg , J. Björk , C.‐G. Elinder , A. Grubb , I. Mejare , et al. 2014 Measuring GFR: a systematic review. Am. J. Kidney Dis. 64:411–424. 24840668 10.1053/j.ajkd.2014.04.010

[phy213205-bib-0021] Sturgeon, C. , A. D. Sam , and W. R. Law . 1998 Rapid determination of glomerular filtration rate by single‐bolus inulin: a comparison of estimation analyses. J. Appl. Physiol. 84:2154–2162. 9609812 10.1152/jappl.1998.84.6.2154

[phy213205-bib-0022] White, C. A. , D. Huang , A. Akbari , J. Garland , and G. A. Knoll . 2008 Performance of creatinine‐based estimates of GFR in kidney transplant recipients: a systematic review. Am. J. Kidney Dis. 51:1005–1015. 18455847 10.1053/j.ajkd.2008.02.308

