# Supplementary material for: Validation of a routine two‐sample iohexol plasma clearance assessment of GFR and an evaluation of common endogenous markers in a rat model of CKD
Source: Physiol Rep. 2017 May 8;5(9):e13205. doi: 10.14814/phy2.13205 (PMC5430119; doi:10.14814/phy2.13205)
Supplement: Supplementary file 1 — Table S1. Log‐linear slope for determining sample time points for one‐compartment analysis. [file PHY2-5-e13205-s001.docx]

**Table S1: Log-Linear Slope for Determining Sample Time Points for 1-Compartment Analysis**

| **Time Points** | **2-5** | **5-10** | **10-20** | **20-30** | **30-60** | **60-90** | **90-120** | **120-180** | **180-240** |
| --- | --- | --- | --- | --- | --- | --- | --- | --- | --- |
| Slope of Inulin Data Points | -0.151 ± 0.041 | -0.076 ± 0.035 | -0.044 ± 0.016 | -0.023 ± 0.009 | -0.023 ± 0.028 | -0.016 ± 0.012 | -0.014 ± 0.011 | -0.012 ± 0.009 | -0.011 ± 0.007 |
| Slope of Iohexol Data Points | -0.069 ± 0.038 | -0.031 ± 0.024 | -0.018 ± 0.0090 | -0.019 ± 0.012 | -0.016 ± 0.009 | -0.015 ± 0.010 | -0.017 ± 0.012 | -0.012 ± 0.012 | -0.013 ± 0.011 |

The natural logarithm of values was taken, then the slope between values was generated to determine when the log-linear phase of decline has shifted. Slope was calculated as ln(value time 1)-ln(value time 2)/time 1-time 2. Slope analysis reveals that past 30 minutes the log-linear slope is similar; thus this time point is used as the earliest possible time point for 1-compartment analysis.
